# Working area effects on the energetic distribution of trap states and charge dynamics of dye-sensitized solar cells†

**DOI:** 10.1039/c8ra09330j

**Published:** 2019-01-14

**Authors:** Wei Yan, Ming-Ming Huo, Rong Hu, Yong Wang

**Affiliations:** Laser Research Institute, Qilu University of Technology (Shandong Academy of Sciences) Qingdao Shandong 266100 China weiyan@sdlaser.cn huo_mingming@126.com yongwang@sdlaser.cn; Research Institute for New Materials Technology, Chongqing University of Arts and Sciences Chongqing 402160 China China.hurong_82@163.com

## Abstract

Measuring the transient photoelectric signals (photovoltage or photocurrent) after optically perturbing dye-sensitized solar cells (DSSCs) can provide information about electron transport and recombination. Herein, the energetic distribution of trap states in different working areas of DSSCs (0.16 cm^2^*vs.* 1 cm^2^) and their impacts on charge transport and recombination were investigated by means of time-resolved charge extraction (TRCE), transient photovoltage (TPV) and transient photocurrent (TPC) measurements. The results indicated that increasing the working area deepened the energetic distribution of trap states (*i.e.*, increased the mean characteristic energy *k*_B_*T*_0_), which hindered the electron transport within the photoanode, accelerated the electron recombination in high voltage regions, and reduced the charge collection efficiency. All abovementioned are the inherent reasons why the *J*_SC_ in larger working area cells is significantly smaller than that in smaller area cells (11.58 mA cm^−2^*vs.* 17.17 mA cm^−2^). More importantly, as the investigation of high-efficiency large area solar cells is currently a promising research topic for new solar cells, we describe the importance of photoanode optimization to achieve high-efficiency DSSCs with large working area by improving charge collection efficiency.

## Abstract 2

The larger working area will deepen the energetic distribution of traps states of dye-sensitized solar cells.

## Graphical abstract

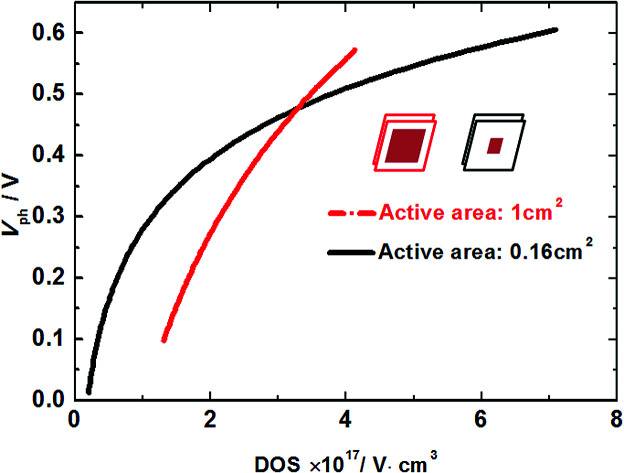

## 1. Introduction

Electron transport dynamics and recombination kinetics are major determinants of the overall efficiency of dye-sensitized solar cells (DSSCs). DSSCs consist of a dyed semiconductor photoelectrode permeated with a redox electrolyte. Following electron injection of the photoexcited dye molecules (attached to the semiconductor nanoparticle surface) into the semiconductor conduction band (cb), the electrons travel through the TiO_2_ film. These electrons are collected at the back contact unless they recombine with the redox species in the electrolyte (e^−^ + S^+^ → S) or recombine with the oxidised dye molecules bound to the semiconductor surface (e^−^ + Ox → Red).^1–3^ Because of the rapid regeneration of the oxidized dyes by the electrolytes, charge recombination between the oxidised dye molecules and the electrons in the photoanode is often negligible, and recombination between the electrons in the TiO_2_ film and the acceptors in the electrolyte is regarded as the dominant pathway of charge recombination.^4–7^

The transport of the injected electrons to the collecting electrode occurs primarily by diffusion, and the transport dynamics have been explained by assuming trap-limited transport to involve an exponential distribution of localized trap states adjacent to the conduction band edge.^4,8^ The recombination and diffusive transport of charges are competitive with each other. Thus, if the recombination is rapid relative to the transport, the fraction of injected electrons collected is reduced, thereby reducing the photocurrent.^9,10^ The rate of electron recombination can also limit the maximum photovoltage since the electron concentration of the substrate primarily determines the photovoltage.^11^ So, the overall efficiency of cells are largely determined by the electron transport and recombination kinetics, and the study of those kinetics in DSSCs is of great significance for improving the structure and materials of DSSC photoanodes, the short-circuit current density, and the PCE of cells.

To investigate the chemical and physical properties of DSSCs, a small-scale laboratory cell is usually fabricated. However, practical applications such as functional windows and tiles for building integrated photovoltaics require larger scale area cells.

A large working area usually reduces the PCE of DSSCs; however, the underlying mechanism is still not fully understood. It is believed that expanding the area increases the *R*_FTO_ (FTO substrate resistance), resulting in a higher series resistance. However, non-uniform morphology and electrode thickness are bottlenecks in fabricating large scale DSSC.^12^ For example, for three cells reported by Park *et al.*, the TiO_2_ film areas were 4 × 4 mm, 5 × 5 mm and 6 × 6 mm and the corresponding efficiencies were 7.9%, 7.4% and 6.6%, respectively.^13^ A PCE of 5.52% was achieved in a 5 × 5 cm active area device, which is 10.4% lower than a small-sized cell (0.6 × 0.6 cm) prepared at similar conditions by W. J. Lee.^14^ The T-1 and T-2 cells used herein (Table 1) have a 6-fold difference in area, resulting in a 44% reduction in PCE. In addition to the increase in the *R*_FTO_, we demonstrate that the anode area strongly affects the DOS distribution, electronic transport/recombination kinetics and charge collection efficiency.

**Table 1:** Short-circuit currents (*J*_SC_), open-circuit voltages (*V*_oc_), filling factors (FF) and power conversion efficiency (PCE) of the DSSCs based on different active areas

DSSC		Device parameters			
Serial number	Area	*J*_SC_ (mA cm^−2^)	*V*_oc_ (V)	FF (%)	PCE (%)
T-1	0.16 cm^2^	17.17	0.72	0.69	8.54
T-2	1 cm^2^	11.58	0.67	0.61	4.79

Further, we used optoelectronic (photovoltage and photocurrent) transient and time-resolved charge extraction (TRCE) measurements to detect the internal electron transport, recombination and distribution of trap state density for two DSSCs with different working areas. Optoelectronic (photovoltage and photocurrent) transient and charge extraction measurements are very useful tools for understanding transient processes occurring in DSSCs. The data from photovoltage (TPV) and photocurrent (TPC) transient measurements can provide information about the transport and recombination of charge carriers in a device. The data from charge extraction can provide information about the charge concentration stored in TiO_2_ under operational conditions. The results indicate that increasing the working area deepens the distribution of trap states and increases the mean characteristic energy *k*_B_*T*_0_, which may accelerate the recombination of electrons and reduce the electronic collection efficiency. The above factors explain why the *J*_SC_ of larger working area cells is significantly smaller than that of smaller area cells (11.58 mA cm^−2^*vs.* 17.17 mA cm^−2^). This result highlights the importance of improving charge collection efficiency to achieve high-efficiency DSSCs with a large area. This improvement can be achieved by modifying the photoanode morphology,^15^ electrolyte engineering,^16^ as well as other approaches.

## 2. Materials and methods

Dye-sensitized TiO_2_ cells were purchased from China Yingkou OPV Tech New Energy CO., LTD. The substrate was transparent conductive FTO glass. Layers of the TiO_2_ particles (10–20 μm) were deposited by screen printing. The TiO_2_ film thickness was 14.8 μm, measured with a profilometer (Tokyo Seimitsu Co., Surfcom 130 A) and areas were 0.4 × 0.4 cm^2^ for T-1 and 1 × 1 cm^2^ for T-2. The dye was N719. The counter electrode was platinized FTO. The electrolyte was a mixture of I_2_ (0.07 M), LiI, 1-propyl-3-methylimidazolium iodide (PMII), guanidinethiocyanate (GuNCS) and *tert*-butylpyridine in an acetonitrile solvent.

**Fig. 1:**
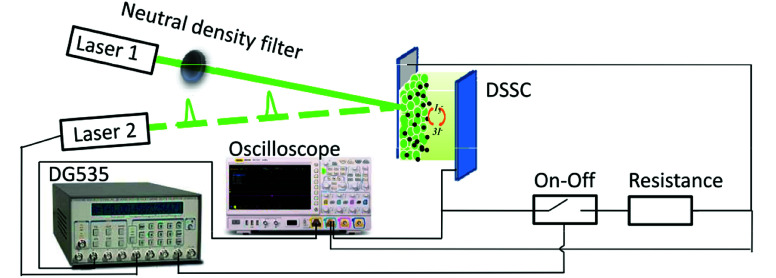
Schematic layout of optoelectronic (photovoltage and photocurrent) transient and time-resolved charge extraction measurement setups.

The apparatus shown in Fig. 1 consists of two green lights (530 nm) laser diode: one irradiated a continuous-wave (laser 1); one modulated by a delay pulse generator (DG535, Stanford Research Systems) irradiated a pulse-wave (laser 2). An electrical analogy switching unit was set in serial connection to a sampling resistor, which was set as a whole in parallel connection to the DSSC device.^15^ A digital oscilloscope is also included.

**Fig. 2:**
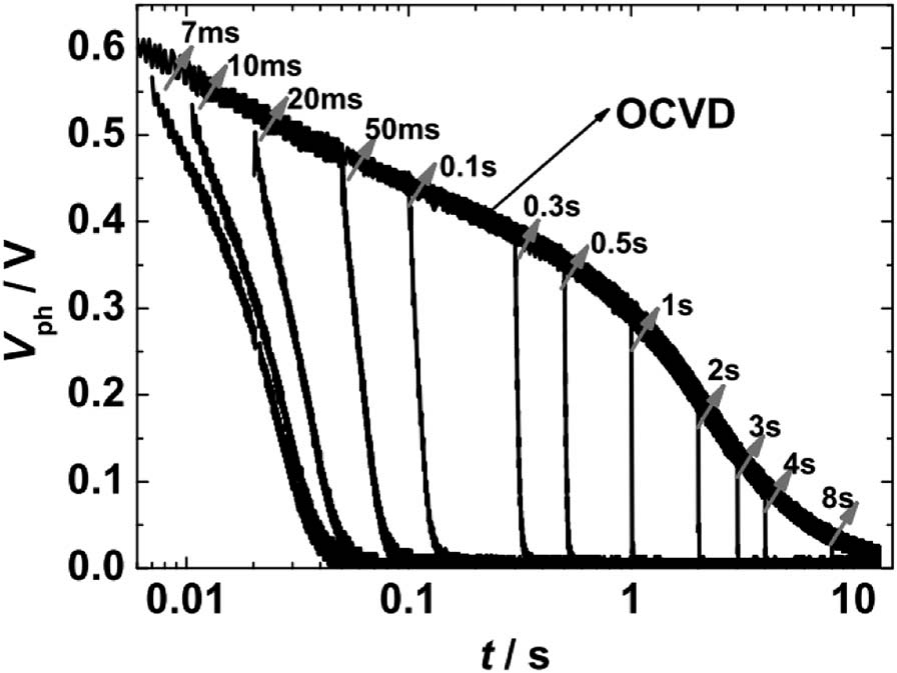
Representative kinetic traces of the overall photovoltage (*V*_ph_) for a DSSC device under open circuit conditions, and the charge extraction kinetics at the indicated times recorded by switching the DSSC to a short circuit condition.

For TRCE measurements, it is needed to record the open-circuit voltage decay (OCVD) curves at first. The target DSSC was kept under open-circuit conditions (turn off laser 1 and disconnect electrical switching) and was irradiated by pulse laser (laser 2, 5 ms, 0.1 Hz) to generate the photovoltage (*V*_ph_). The decay profiles of *V*_ph_ was recorded by the oscilloscope, as shown in Fig. 2. Then, by the utilization of the fast switch unit to quickly switch the measurement system from open- to short-circuit at a desired timing within the decay of *V*_ph_, the kinetics of charge extraction can be obtained (Fig. 2). The switching-timing was regulated by DG535. TRCE measured the integrated photocurrent after switching off a light source at various time delays before switching the cell to short circuit. The technique was used to directly demonstrate an exponential dependence between the concentration of charges in the device and the *V*_ph_. This allowed experimental confirmation of the multiple trapping model as a means to describe both electron transport and recombination.

For TPV measurements, the target DSSC was kept on open-circuit conditions (disconnect electric switching) and was irradiated by a continuous-wave (laser 1) to maintain a desired *V*_ph_. Placing neutral density filters into the illumination path caused a systematic change in the initial quasi-equilibrium conditions. A perturbation pulse wave (laser 2, 1 μs, 0.1 Hz) was then applied to induce a small increase in *V*_ph_, (Δ*V*_ph_/*V*_ph_ = 5%). The electric signals were recorded by the oscilloscope (shown in Fig. 5). When using a resistor to short the test circuit, the TPC kinetics can be detected by the oscilloscope.

## 3. Results and discussion

### 3.1. Trap state DOS and spatial distribution as revealed by TRCE

**Fig. 3:**
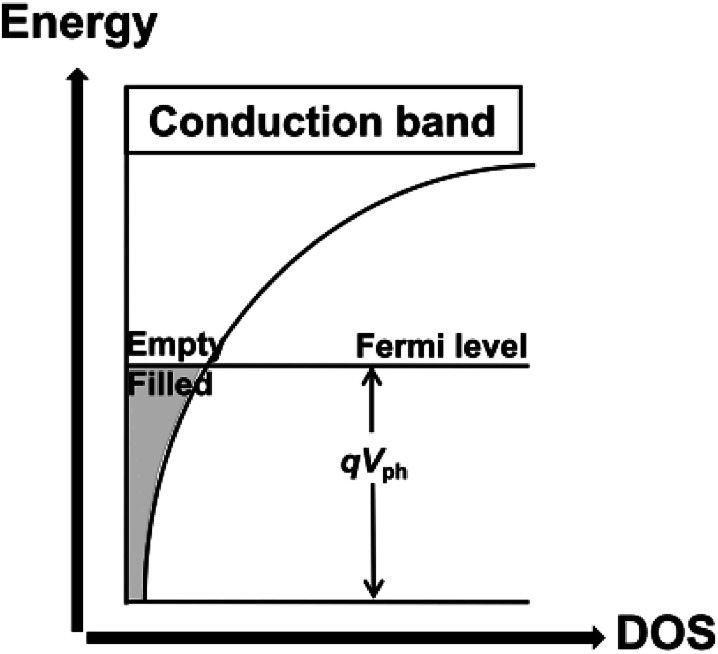
Illustration of the dependence of the trapped electron density on the photovoltage for an exponential distribution of traps.^17^

The density of trap states (DOS) between Fermi levels in the dark (*E*_f,redox_) and under illumination (*E*_f,redox_ + *eV*_ph_) and the trap distribution parameter *β* can be directly detected by TRCE. The basic principle of TRCE is described in the Materials and methods section. The relationship between the photovoltage and the density of the extracted electrons is shown in Fig. 3.^17^ The electron trap occupancy is determined by Fermi–Dirac statistics. The experimentally observed distribution was exponential, but other forms of trap distribution also exist.^18^

**Fig. 4:**
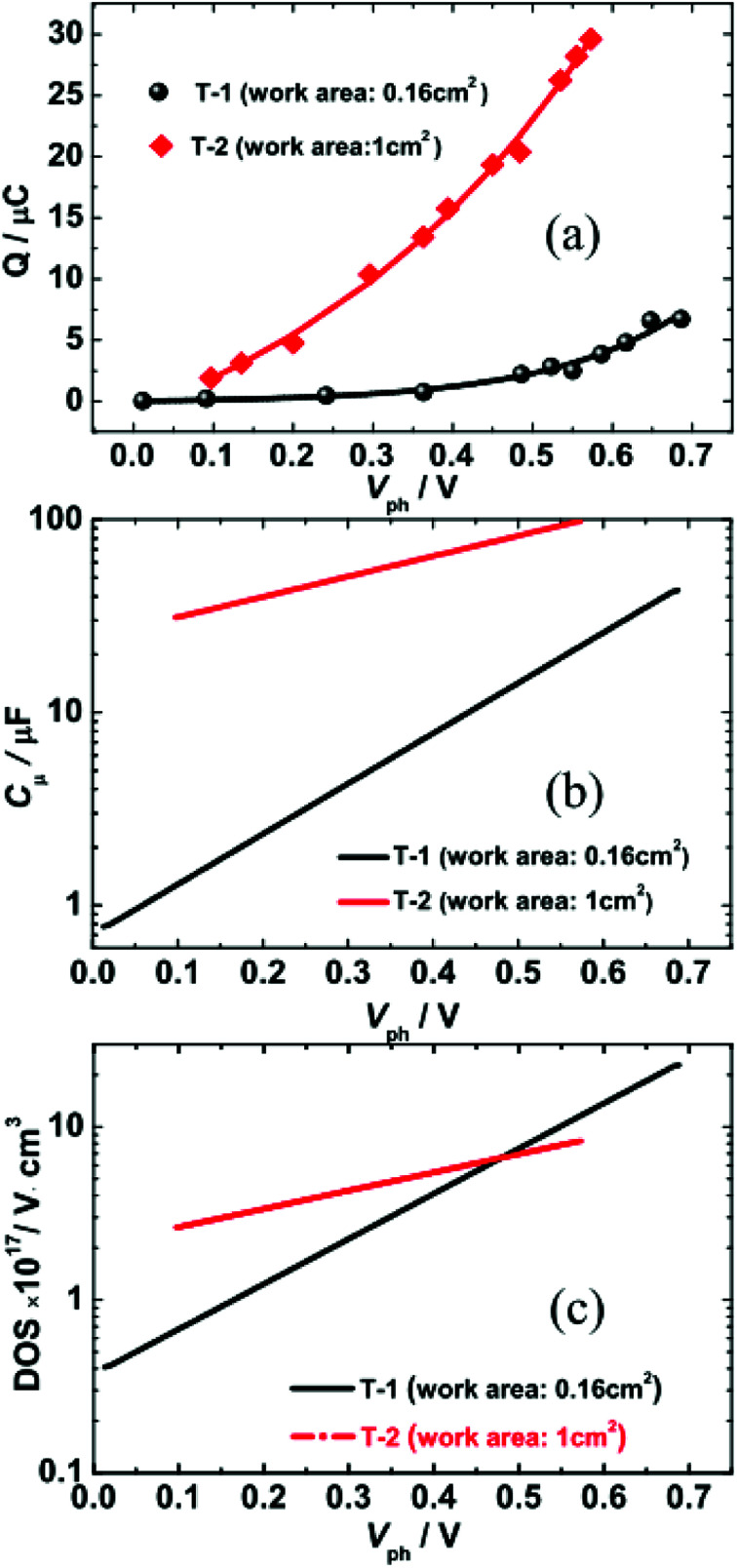
(a) Extracted charge as a function of photovoltage (*V*_ph_). (b and c) Semi-logarithmic plots of chemical capacitance *C*_μ_ (b) and DOS (c) against *V*_ph_ for T-1 and T-2 solar cells.

Fig. 4(a) illustrates the total charge (*Q*) extracted at different values of *V*_ph_ from the two DSSCs. Each *Q*–*V*_ph_ exhibits nonlinear increasing phases and can be well described by a monoexponential function. On the basis of eqn (1):1
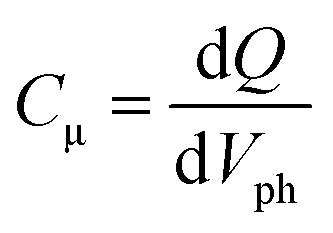
where *C*_μ_ is the chemical capacitance, which is widely adopted as a metric of the DOS of semiconducting TiO_2_ photoanodes.^19^ We derived the *C*_μ_–*V*_ph_ dependence for the two cells by taking the first derivation of the fitted curves from Fig. 4(a) and further deriving the DOS–*V*_ph_ dependence using eqn (2):^4,15^2
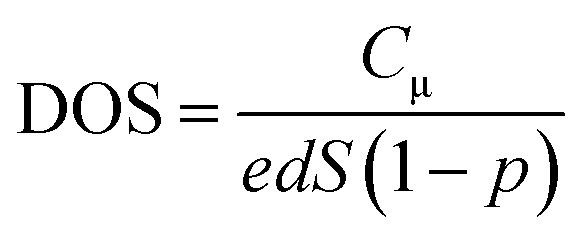
Here, *e* is the unity charge, *d* is the film thickness, *S* is the effective area of the photoanode, and *p* is the porosity of the TiO_2_ layer (assumed to be 50%). The obtained *C*_μ_–*V*_ph_ and DOS–*V*_ph_ dependences are shown in Fig. 4(b) and (c).

In Fig. 4(b), the *C*_μ_ is exponentially dependent on *V*_ph_, and the dependence can be theoretically rationalized using eqn (3):^20^3
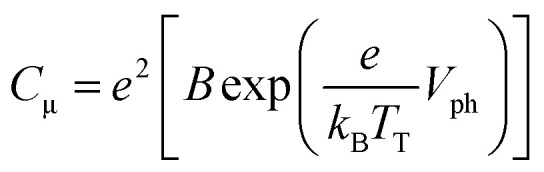
The detailed theoretical derivation of eqn (3) can be found in the ESI.† Here, *B* is the pre-exponential factor, *k*_B_ is the Boltzmann constant, and *T*_T_ is the characteristic temperature for the traps. Therefore, *k*_B_*T*_T_ is the characteristic energy for the traps. By fitting the *C*_μ_–*V*_ph_ data (Fig. 4(b)), we found that *C*_μ_(T-1) = 0.723 exp(*V*_ph_/0.166) and *C*_μ_(T-2) = 24.47 exp(*V*_ph_/0.411). The characteristic energies for the traps of the two cells were 166 meV and 411 meV for T-1 and T-2, respectively. The *k*_B_*T*_T_ value increases with the increase in the working area of the cell, which indicates the photoanode area dependence of the DOS distribution. The observed increase in *k*_B_*T*_T_ suggests that the trap states deepen.

As shown in Fig. 4(c), the DOS is also exponentially dependent on *V*_ph_. This dependence is generally attributed to the exponential distribution of delocalised trapping states below the conduction band edge that can accept electrons. The dependence can be theoretically rationalized according to eqn (4):^17,21^

4 *N*(*E*) = *N*(*eV*_ph_) = *N*(0)exp[*βeV*_ph_/*k*_B_*T*]

Here, *N*(*E*) is the density of the extracted electrons, *N*(*0*) is the value of the density of states function at the dark Fermi level, and *β* is a parameter that can measure how rapidly the trap density varies with energy. By fitting the DOS–*V*_ph_ data, *β* can be obtained, and was found to be 0.157 for T-1 and 0.063 for T-2. The trap density of T-1 (small working area) changes approximately 2.46-fold more rapidly with energy than that of T-2. Compared with T-1, more trap states are distributed in lower energy locations in T-2, suggesting that more photogenerated charges are located in deeper trap states. Since the rate of thermal detrapping depends on the trap depth, deep traps are difficult to collect and may hinder electron transport within the photoanode network, thus reducing the *J*_SC_ (see next section for details).

### 3.2. Charge recombination and collection as revealed by TPV and TPC

**Fig. 5:**
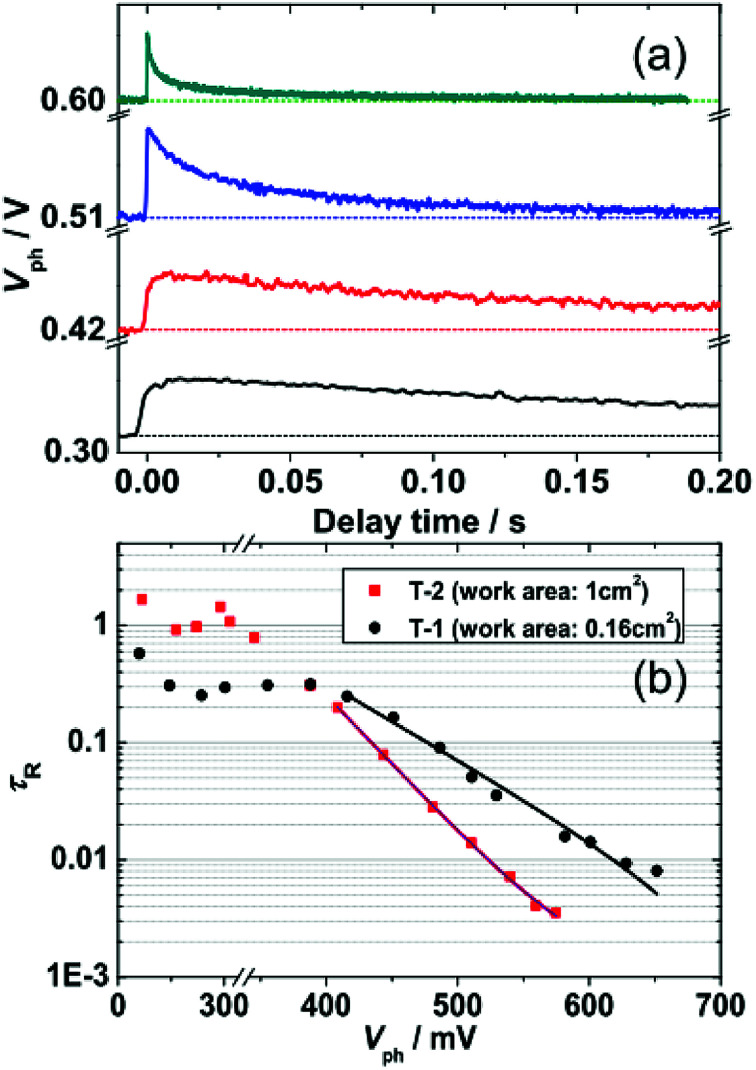
(a) Representative kinetic traces of transient photovoltage (TPV) at specific *V*_ph_. (b) Semi-logarithmic plots of the TPV-determined *τ*_R_ as a function of *V*_ph_ for T-1 and T-2. Solid lines were derived by fitting the data from *V*_ph_ > 400 mV to an exponential model function [eqn (5)].

To assess the effect of working area of DSSCs on trap state DOS and spatial distribution, we further investigated electron collection and the kinetics of electron recombination/transport by TPV and TPC. The experimental details for TPV and TPC can be found in the Methods section. TPV and TPC are belong to small perturbation transient measurements, they are easier to work with because the background concentration of charges in the device remains approximately constant. As a result, single values related to the transport and recombination of electrons in the cell can be obtained for a given steady state condition. Repeated measurements of a cell under different steady state conditions provide a complete picture of the cell. Fig. 5(a) shows an example of the photovoltage decay under different *V*_ph_. The decay of Δ*V* under different *V*_ph_, depends on the rate of electron recombination.^22^ Thus, the electronic recombination time constant *τ*_R_ can be extracted by fitting the decays under different *V*_ph_ to a single exponential function. Fig. 5(b) shows the semi-logarithmic plots of *τ*_R_ and *V*_ph_. For *V*_ph_ < 400 mV, the *τ*_R_, whose value is 0.3 s and 1 s for T-1 and T-2, respectively, is rarely affected by *V*_ph_. This phenomenon may be due to the dominance of surface-trap isoenergetic charge recombination.^15,20^ For *V*_ph_ > 400 mV, *τ*_R_ is exponentially dependent on *V*_ph_, which is due to the limited multiple-trap charge recombination.^4^ In the region of 400–600 mV, the *τ*_R_ of T-2 (large working area) are shorter than those of T-1 (small working area). Because of the rapid regeneration of oxidized dyes by electrolytes (<a few microseconds), the electron recombination with dye cations (e^−^ + *S*^+^ → *S*) will be negligible in TPV measurements throughout this paper. The recombination time constant *τ*_R_ is only related to the redox electrolyte (e^−^ + Ox → Red). There is a relationship between the *τ*_R_ and the *V*_oc_ (Table 1), that is, the lower values of *V*_oc_ for T-2 is due to the shorter *τ*_R_.^23^

Fitting the *τ*_R_–*V*_ph_ data in the 400–600 mV range to eqn (5)^15,24,25^ can yield the characteristic *k*_B_*T*_0_ energies of 43 meV for T-1 and 80 meV for T-2.5
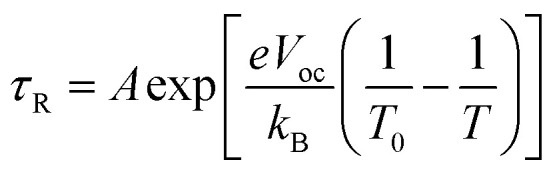
Here, *A* is a pre-exponential factor and *k*_B_*T*_0_ represents a mean characteristic energy for evaluating trap states involved in charge recombination. As *V*_ph_ increases, the electronic recombination rate of larger working area DSSC (T-2) increases relative to that of the small work area cell (T-1). This effect is attributed to the deeper DOS distribution (larger characteristic energy *k*_B_*T*_0_). These results are consistent with the DOS distribution revealed by TRCE.

When the electrons in a photoanode populate deeper trap states, excitation into conduction bands becomes more difficult. Hence, hopping among the trap states is suggested to be the dominant pathway of electron transport. Electrons will recombine with the electrolyte predominantly through the surface trap states. The surface traps are localized electronic states in the band gap and are physically located either at the TiO_2_ surface or within a tunneling distance from the surface.^16^ As the electrons trapped by surface states are intensely localized, the electron transfer from TiO_2_ to electrolyte is slower, *i.e.*, the recombination channel is faster.^20^ A deeper DOS distribution indicates that more electrons are in deep trap states; thus, the electronic recombination rate of the large working area DSSC (T-2) is faster than that of the small working area cell (T-1).

Under a specific *V*_ph_, repeating the experiment after short-circuiting the test loop of TPV results in a transient photocurrent (TPC). The pulse causes a perturbation of the electron distribution inside the TiO_2_ photoanode, which causes a small current to flow through the external circuit. Therefore, the pulse intensity only requires <10 mV shift in the Fermi level, the resulting current transients measure the transport of electrons that occur at a given *V*_oc_. By exponential fitting of the decay of the current transients, the electron collection time constant (*τ*_C_) can be obtained. By integration, the charge transient can also be obtained.^26^ The approximate electron collection efficiency (*η*_cc_) can be obtained through eqn (6).^27–29^6
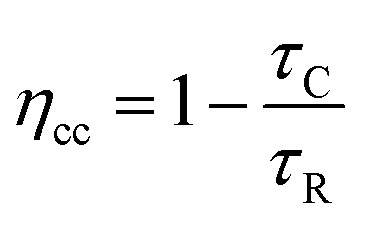


**Fig. 6:**
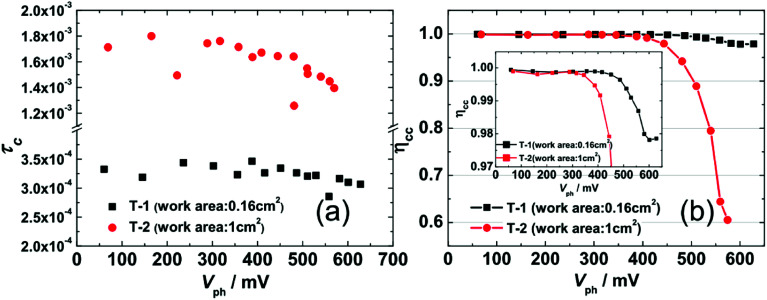
(a) The TPC-determined electron collection time constant *τ*_C_ as a function of *V*_ph_ for T-1 and T-2. (b) eqn (6)-determined electronic collection efficiency *η*_cc_ as a function of *V*_ph_ for T-1 and T-2. Inset: to show the *η*_cc_ of T-1 in detail, the region from 0.97 to 1 on the *y*-axis has been enlarged.

Fig. 6(a) shows the TPC-determined *τ*_C_ as a function of *V*_ph_ that were obtained by fitting current decays. Firstly, the value of *τ*_C_ for T-1 (0.25–0.35 ms) is an order of magnitude smaller than that for T-2 (1.3–1.8 ms), which is consistent with the deeper DOS distribution of T-2 and it hinders the electron transport within the photoanode network. Secondly, similar with *τ*_R_, *τ*_C_ is rarely affected by *V*_ph_ in the region of *V*_ph_ < 400 mV; however, when *V*_ph_ > 400 mV, the *τ*_C_ slightly decreases with an increase in *V*_ph_, implying that the electron transport dynamics also obey the multiple-trap mechanism. The *η*_cc_ shown in Fig. 6(b) indicates that T-1 is close to 1 in the range of the measured *V*_ph_, while the *η*_cc_ for T-2 drops significantly in the range of *V*_ph_ ∼ (450–600 mV). Thus, the lower *J*_SC_ (11.58 mA cm^−2^, Table 1) for the larger-area cell (T-2) is due to its lower collection efficiency.

The results described in Sections 3.1 and 3.2 reveal that large working area cells require TiO_2_ film optimization to control of the DOS distribution and increase the charge collection efficiency. UV exposure and HCl treatment of TiO_2_ films are potentially good methods for accomplishing this optimization. UV exposure reversibly creates a high concentration of photoactive surface states; these states were described to be continuously distributed below the conduction band edge. As shallow electron traps, these would be beneficial for electron injection from the dye and transport by the thermally activated detrapping process.^30,31^ Acid treatment increases the density of protonated sites and favors multidentate dye adsorption.^30,31^ In addition, the crystal structure of TiO_2_ and nanoparticle sizes are important for the optimization of films. Photoanode films with the amorphous TiO_2_ removed exhibit a higher density of shallow traps that receive more electrons generated from the excited dye and increased *J*_SC_.^32^ Increasing the TiO_2_ particle size can lead to energetically shallower trap states, which would increase the values of *J*_SC_. However, a larger TiO_2_ particle size reduces the number of deep trap states because of the relatively smaller internal surface area. Shrinking the internal surface area of the photoanode will also depress the overall dye*-to-TiO_2_ electron injection owing to the diminished number of adsorbed dye molecules, which in turn reduces the *J*_SC_.^15^ It is a competitive process. Thus, the optimization of photoanode films with specific materials and working areas requires a careful consideration of the advantages and disadvantages of various methods, which needs further experimental verification.

## 4. Conclusions

The distribution of electronic trap states for dye-sensitized solar cells with different working areas was determined by time-resolved charge extraction. By comparing the *β* parameter (0.157 for T-1 and 0.063 for T-2), the trap density of small working area cells were found to change more rapidly with energy by a factor of 2.46 than those of larger working area cells, implying that the former possesses an energetically shallower DOS distribution profile. TPV and TPC measurements indicated that the larger working area of cells increases the recombination rate while slowing the transport rate; this effect was attributed to the larger characteristic energy *k*_B_*T*_0_ (80 meV *vs.* 43 meV for T-2 and T-1, respectively), *i.e.*, the deeper DOS distribution. The results were consistent with the DOS distribution revealed by TRCE. These results summarily explain why the *J*_SC_ of larger working area cells is significantly smaller than that of smaller area cells (11.58 mA cm^−2^*vs.* 17.17 mA cm^−2^). The correlation between the energetic distributions of DOS, the dynamics of charge recombination/transport and the device performance of different work areas revealed that large working area cells require control of the DOS distribution and an increase in the charge collection efficiency by optimizing TiO_2_ films. This optimization can be accomplished by UV exposure and HCl treatment of TiO_2_ films, removal of amorphous TiO_2_, and increasing the TiO_2_ particle size.

## Conflicts of interest

There are no conflicts to declare.

## Supplementary Material

RA-009-C8RA09330J-s001
