# Supplementary material for: Working area effects on the energetic distribution of trap states and charge dynamics of dye-sensitized solar cells
Source: RSC Adv. 2019 Jan 14;9(3):1734–40. doi: 10.1039/c8ra09330j (PMC9059758; doi:10.1039/c8ra09330j)
Supplement: RA-009-C8RA09330J-s001 [file RA-009-C8RA09330J-s001.pdf]

Electronic Supplementary Material (ESI) for RSC Advances.
This journal is © The Royal Society of Chemistry 2018

Supplementary information

Working Area Effects on the Energetic Distribution of Trap States and Charge Dynamics of Dye-Sensitized Solar Cells

Wei Yan[a], Ming-Ming Huo*,[a], Rong Hu[b], Yong Wang[a]

[a], Laser Research Institute, Qilu University of Technology (Shandong Academy of Sciences), Qingdao, Shandong, 266100, China;

[b], Research Institute for New Materials Technology, Chongqing University of Arts and Sciences, Chongqing, 402160, China.

S1.Theoretical derivation of chemical capacitance

When the quasi-Fermi level (E_{Fn}) of a dye-sensitized solar cell is much lower than the band edge energy of conduction band (E_{cb}) of its TiO_2 photoanode, the chemical capacitance (C_μ) is dominated by the trap state distribution. C_μ can be written as[S1]:

$$C_\mu = e^2 \frac{\partial n_T}{\partial E_{Fn}} \tag{s1}$$

where e is the unit charge, n_T is the density of trapped electrons. At this time, the chemical capacitance reflects the capability of a system to accept or release additional trapped electrons with density n_T due to a change of quasi-Fermi level.

n_T can be written as[S2]

$$n_T = \int_{E_{vb}}^{E_{cb}} g_T(E) f(E, E_{Fn}) \mathrm{d}E = N_T \exp\left[\left(E_{Fn} - E_{cb}\right)/ k_B T_T\right] \tag{s2}$$

Where E_{vb} is the band edge energy of the valence band, $g_T(E)$ is the density of trap states as a function of energy E, $f(E, E_{Fn})$ is the Fermi-Dirac function, N_T and T_T are the total density and the characteristic temperature of the trap states.

For trapped electrons in Boltzmann distribution, C_μ can be expressed as[S3]

$$C_\mu = e^2 \frac{N_T}{k_B T_T} \exp \frac{E_{Fn} - E_{cb}}{k_B T_T} \tag{s3}$$

Because of $V_{ph} = (E_{Fn}$-$E_{redox})/e$, E_{redox} is the redox energy of electrolyte. Eq. (s3) can be expressed as

$$C_\mu = e^2 \left[B \exp\left(\frac{e}{k_B T_T} V_{ph} \right) \right] \tag{s4}$$

So C_μ is exponentially dependent on V_{ph}. The pre-exponential factors B is

$$B = \frac{N_T}{k_B T_T} \exp\left(\frac{E_{redox} - E_{cb}}{k_B T_T} \right) \tag{s5}$$

References

[S1] Y. Wang, D. Wu, L.-M. Fu, X.-C. Ai, D. Xu and J.-P. Zhang, *Chem. Phys. Chem*. 2015, **16**, 2253.

[S2] J. Bisquert, V. S. Vikhrenko, *J. Phys. Chem. B* 2004, **108,** 2313.

[S3] J. Bisquert, A. Zaban, M. Greenshtein and I. M. Seró, *J. Am. Chem. Soc.* 2004, **126**, 13550.
